# Surgical Pathology

**Published:** 1884-09

**Authors:** 


**Surgical Pathology.** By A. J. PEPPER, M. B., M. S., F. R. C. S., Surgeon and Lecturer at St. Mary's Hospital, London.

